# Diet as a Risk Factor for Rheumatoid Arthritis

**DOI:** 10.7759/cureus.39273

**Published:** 2023-05-20

**Authors:** Chelsea M Guan, Shazia Beg

**Affiliations:** 1 Internal Medicine Residency, University of Central Florida Hospital Corporation of America (HCA) Healthcare Graduate Medical Education (GME), Orlando, USA; 2 Internal Medicine Residency, University of Central Florida College of Medicine, Orlando, USA; 3 Rheumatology, University of Central Florida College of Medicine, Orlando, USA

**Keywords:** autoimmune, rheumatoid arthritis, risk factor, nutrition, diet

## Abstract

Rheumatoid arthritis (RA) is a chronic, systemic inflammatory disease that involves primarily synovial tissues and typically affects women more than men. An exact cause has yet to be identified, but the disease is thought to manifest due to both genetic and environmental factors. The predominant theory is that RA is an autoimmune disease with environmental triggers. Recently, diet as a risk factor for RA has become of interest. The objective of this narrative review is to determine which dietary factors have an influence on developing RA by examining existing literature on this topic. A PubMed search was built using the MeSH terms: “rheumatoid arthritis,” “risk factors,” “diet,” “nutritional status,” “nutrition therapy,” “nutrition assessment,” “nutrition disorders,” “diet, food, and nutrition,” and “nutritional requirements.” Articles containing a sample size of >10, published in the last 30 years, and written in English were included. Current literature has examined dietary items, such as alcohol, fruit, red meat, and caffeinated beverages, as risk factors for RA. However, the effect of each dietary item has often been variable across studies. The variation in results may be attributed to the variable categorization of each dietary item across studies, variations in the phrasing of dietary items, differing methods of data collection, and the cohort chosen. This narrative review of the literature showed that moderate alcohol consumption and increased β-cryptoxanthin are protective against developing RA. Overall, specific dietary elements and their influence on RA risk is a promising topic, and significant findings may be helpful in preventing the development of RA.

## Introduction and background

Rheumatoid arthritis (RA) is a chronic, systemic inflammatory disease that involves primarily synovial tissues and typically affects women more than men [1]. If left untreated, the disease may cause joint damage, cardiovascular complications, and other complications. Uncontrolled RA may lead to poor quality of life. While an exact cause has yet to be identified, the disease is thought to manifest due to both genetic and environmental factors, namely smoking [2]. The influence of diet as a risk factor for RA has become of increased interest in recent years. Dietary factors are a potential modifiable risk factor for developing RA. However, it is still unclear which dietary factors have an influence on developing RA, and what those influences are. There have been multiple studies done regarding the impact of diet as a risk factor for RA, but the results have often been mixed. This narrative review focuses on clarifying the influence of diet as a risk factor for RA. This article was previously presented as a meeting poster and abstract at the 2019 ACR/ARP Annual Meeting on November 10, 2019 [3].

## Review

Methods

PubMed was searched for relevant articles. Articles that contained a sample size of >10, published in the last 30 years, and written in English were included. A search was built only using the following MeSH terms: “rheumatoid arthritis,” “risk factors,” “diet,” “nutritional status,” “nutrition therapy,” “nutrition assessment,” “nutrition disorders,” “diet, food, and nutrition,” and “nutritional requirements.” Each MeSH search included “rheumatoid arthritis,” “risk factors,” and one of the aforementioned terms. The abstracts were first screened for the aforementioned inclusion criteria, and suitable articles were reviewed. Studies focusing on vitamins and supplements were excluded. Additional relevant articles were found from the references of reviewed articles. A total of 127 articles were reviewed and 50 were included. Because the categorization of each dietary item varied across studies, it was important to examine the details carefully and to ensure consistency among categorizations before analyzing the data. For example, alcohol intake may refer to only wine, only beer, and both wine and beer depending on the paper. Some studies reported results on RA risk based on whether the RA was seropositive or seronegative, variations in phrasing/definitions (e.g., “fruits” sometimes included citrus, other times it did not, and sometimes it was not defined at all), methods of data collection, and cohort chosen.

Results

Alcohol

Alcohol, consumed by many people regularly in many forms, such as wine, beer, or liquor, has been shown to have anti-inflammatory effects when consumed in moderate amounts ( one to two drinks a day for women and men, respectively) [4]. Thus, it has been hypothesized to reduce the risk of RA.

A case-control study of a Netherlands population demonstrated that moderate alcohol consumption (one to two drinks a day versus zero drinks a day) decreases the risk of developing RA [5]. A frequency-matched case-control study in a Denmark population featuring 515 patients who were recently diagnosed with RA and 769 sex and age-matched controls looked at the influence of various environmental factors on the development of RA. This study showed that increased alcohol (beer, wine, dessert wine, or spirits) consumption (>15 drinks a week versus 0-15 drinks) reduces the risk of developing anti-CCP-positive RA [6]. Furthermore, consumption of six to 10 glasses of wine/week versus zero to five drinks or >10 glasses of wine/week decreases the risk of developing RA [6]. Analysis of data from the Swedish Epidemiological Investigation of Rheumatoid Arthritis (EIRA) and Danish Case-Control Study on Rheumatoid Arthritis (CACORA) case-control cohorts showed an association between increased alcohol consumption and decreased risk of developing RA (P<0.001) [7]. Specifically, RA patients who were in the highest quartile of alcohol consumption had a 40%-50% decreased risk of developing RA in comparison to patients who were in the half with a lower consumption (EIRA, OR=0.5 (95% CI 0.4 to 0.6); CACORA, OR=0.6 (95% CI 0.4 to 0.9)) [7]. Another prospective cohort study featuring 34,141 women from the Swedish Mammography Cohort, which is a population-based cohort from central Sweden featuring women born during 1914-1948, demonstrated that moderate alcohol consumption (>4 glasses of alcohol/week, with a median of six glasses/week, versus zero to four glasses/week) is associated with a decreased risk of developing RA [8].

Similarly, a prospective cohort study featuring the Nurses’ Health Study (NHS), which began in 1976 and features 121,701 female registered nurses in the US, and NHS II, which began in 1989 and features 116,430 female nurses, found an association between long-term moderate alcohol consumption and decreased risk of developing RA [9]. A case-control study featuring 873 white Caucasian patients with RA and 1,004 Caucasian healthy controls showed that increased consumption of alcohol decreases the risk of developing RA (OR of developing RA for no drinks versus alcoholic drinks >10 days a month 4.17 (3.01-5.77)) [10]. Another study featuring the NHS cohort that focused on dietary quality, as evaluated by AHEI-2010 (Alternative Healthy Eating Index) components, demonstrated a significant association between moderate alcohol consumption and decreased risk of developing RA [11]. 

A population-based case-control study featuring 349 participants and 1,457 controls from King County, WA, and Group Health Cooperative of Puget Sound Studies showed an association between >14 weekly alcoholic drinks and decreased risk of developing RA in post-menopausal women (RR=0.5; 95% CI=0.2-1.7) [12]. Another study featuring the NHS cohort also demonstrated an association between low alcohol consumption and an increased risk of developing RA [13]. A nested case-control study using the Malmo Diet and Cancer Study (MDCS), a prospective cohort, which includes 30,447 subjects recruited between 1991 and 1996, also suggested an association between increased alcohol consumption and reduced risk of developing RA (OR of individuals with baseline alcohol consumption of 3.5-15.2 g/day versus <3.5 g/day 0.48 (0.22-1.05)) [14]. In contrast, some studies have also shown that there is no significant association between alcohol consumption and the risk of developing RA [15-17].

Carotenoids

Carotenoids are antioxidant pigments generated by plants that provide them their red, yellow, and orange colors [18]. Carotenoids can be found in various fruits and vegetables, such as carrots, tomatoes, oranges, and watermelon. α-carotene, β-carotene, β-cryptoxanthin, lycopene, lutein, and zeaxanthin have been studied as risk factors for developing RA. A study examining women in the NHS and NHS II cohorts that featured patients with systemic lupus erythematosus (SLE) and RA found no significant association between increased α-carotene, β-carotene, β-cryptoxanthin, lycopene, lutein, and zeaxanthin consumption via diet and supplements and risk of developing RA [19]. A study using the Iowa Women’s Health Study cohort found a significant association between increased β-cryptoxanthin intake and decreased RA risk (women consuming <40 g/day versus >86.9 g/day, RR=0.59, 95% CI 0.39-0.90) [10]. This study also found no significant association with total carotenoids, α-carotene, β-carotene, lycopene, or lutein/zeaxanthin intake [20]. A nested case-control study featuring the European Prospective Investigation of Cancer in Norfolk (EPIC-Norfolk) cohort found an association between increased β-carotene intake and reduced risk of developing inflammatory polyarthritis (IP), such as RA [21]. A later study by the same authors, which was another nested case-control study featuring the EPIC-Norfolk cohort, did not find a significant association between β-carotene intake and RA risk but found that subjects who were in the highest tertiles of zeaxanthin and β-cryptoxanthin intake had a decreased risk of developing IP, in comparison to subjects in the lowest tertile (OR 0.48, 95% CI (0.24, 0.94) for zeaxanthin, and 0.51 (0.25, 1.02) for β-cryptoxanthin, respectively) [22]. This study also found no significant association between lutein or lycopene intake and the risk of developing IP [22].

Carotenoids, particularly β-cryptoxanthin, appear to be protective against developing RA.

Caffeine

Caffeine has been hypothesized to be a risk factor for developing RA. Coffee, which contains high levels of caffeine, has been shown to significantly increase total cholesterol, LDL-C, and triglyceride levels [23]. Studies have examined different caffeine-containing compounds, namely coffee and tea. Results regarding the risk of consuming caffeinated drinks and developing RA have been mixed. 

Studies showing a protective effect of caffeinated drinks include a 2000 study in a Finnish population that examined caffeine as a risk factor for developing RA. It was found that consuming >4 cups of coffee a day versus <3 cups a day increases the risk of developing RF+ RA (RR for >4 cups consumed versus <3 cups 2.2 (1.13-4.27)) [24]. A study by Mikuls et al. focusing on coffee, tea, and caffeine consumption and the risk of developing RA featured the Iowa Women’s Health Study cohort, which is a prospective cohort of 31,336 women ages 55-69, and showed that consuming >4 cups of decaffeinated coffee a day significantly increased risk for developing RA (RR 2.58 (1.63-4.06)) [25]. The same study showed that consuming >3 cups of tea daily, in comparison to not drinking any tea, was also found to be protective against developing RA (RR 0.39, 95% CI 0.16-0.97) [25]. A case-control study featuring 500 patients with RA and 500 healthy controls found that increased consumption of coffee per month reduces the risk of developing RA (one to seven cups of coffee/month (OR=0.44, CI 0.25-0.76), ≥8 cups of coffee/month (OR=0.50, CI 0.28-0.90)) [26]. This study also found that increased monthly green tea consumption reduces the risk of developing RA (OR=0.65, CI 0.45-0.93) [26].

Studies showing that increased caffeine consumption increases the risk of developing RA include a case-control study featuring 515 patients in a Denmark population who were recently diagnosed with RA and 769 sex and age-matched controls, which demonstrated that consuming >10 cups of coffee a day significantly increases the risk of developing anti-CCP+RA (OR for >10 cups per day versus 0 cups per day 2.18 (1.07-4.42)) but not anti-CCP-RA [6]. In addition, a study by Lamichhane et al. featuring the Women’s Health Initiative Observational Study prospective cohort, which features 76,853 older women, found that increased consumption of caffeinated tea was significantly associated with an increased risk of developing RA (HR=1.40 for caffeinated tea consumption versus no tea consumption; CI, 1.01-1.93; P=0.04) [27].

Several studies have also shown no significant association between caffeine consumption and risk of developing RA. A study using the NHS cohort that examined coffee consumption and the risk of developing RA showed that there is no significant association between the amount of caffeinated coffee consumption, decaffeinated coffee consumption, total coffee consumption, and tea consumption with the risk of developing RA [28]. This study also found no significant association between consuming >4 cups of coffee a day and zero cups a day with the risk of developing RA [28]. Furthermore, a study using the EPIC-Norfolk that examined the association of several dietary components and the risk of developing IP showed no significant association between caffeinated coffee or tea consumption and developing IP [17]. Mikuls et al. found no significant association between total coffee consumption, caffeinated coffee consumption, and total caffeine consumption with the risk of developing RA [25]. Lamichhane et al. did not find a significant association between coffee consumption, including amount, caffeinated versus decaffeinated, and filtered/unfiltered, and risk of developing RA [27].

Altogether, it can be said that the influence of caffeine and its various forms (e.g., coffee and tea) as a risk factor for developing RA is mixed. More research is needed to make a definitive conclusion about any of caffeine’s (or any source of caffeine’s) effects on developing RA.

Meat

Meat has been hypothesized to increase the risk of developing RA, possibly through mechanisms involving meat fat and nitrites [29]. A prospective study featuring NHS and NHS II cohorts that examined the long-term dietary quality and risk of developing RA found a significant association between lower red meat consumption and RA risk, independent of BMI and other variables [11]. A case-control study that focused on dietary intake which featured 968 Han Chinese RA patients and 1,037 matched healthy controls found no significant association between red meat consumption and risk of developing RA [30]. A study examining dietary risk factors and developing inflammatory arthritis featuring the EPIC-Norfolk population found that participants with the highest level of red meat consumption (OR 1.9, 95% CI 0.9-4.0) and meat and meat products combined (OR 2.3, 95% CI 1.1-4.9) led to an increased risk of developing IP, such as RA [17]. A prospective cohort study that focused on protein, iron, and meat consumption in the NHS cohort did not find an association between meat consumption (including poultry) and the risk of developing RA [31].

Carbohydrates

High-refined carbohydrate intake, such as in a “Western diet,” has also been studied as a risk factor for inflammatory autoimmune diseases such as RA [32]. A population-based case-control study featuring 324 female RA participants from western Washington and 1,245 healthy controls found no significant association between carbohydrate consumption (as a percentage of total calories or the total number of grams consumed) and risk of developing RA [33]. A nested case-control study using the Västerbotten Intervention Program (VIP) cohort initially found a statistically significant association between the highest tertile consuming a carbohydrate-restricted diet and increased risk of developing anti-CCP+RA, but this association was no longer significant after adjusting for sodium intake [16]. A case-control study featuring 968 Han Chinese RA patients and 1,037 matched healthy controls found that increased consumption of potatoes increased the risk of developing RA (OR 1.160; 95% CI=1.035-1.300, P=0.011) [30]. Another study featuring the NHS cohort found that increased whole grain consumption may have protective effects against developing RA, which the authors attribute to dietary fiber and antioxidant levels [11].

Currently, it is difficult to conclude if carbohydrate intake is a risk factor for developing RA. Further research is needed to make conclusions. Different forms of carbohydrates may have a stronger impact on the risk of developing RA or preventing the development of RA, such as increased potato consumption (possible increased risk) and increased whole grain consumption (possible decreased risk).

Protein

Increased dietary protein has been hypothesized to increase RA risk [34]. 

A study examining dietary risk factors and developing inflammatory arthritis featuring the EPIC-Norfolk population found that increased total protein (OR 2.9, 95% CI 1.1-7.5) led to an increased risk of developing IP, such as RA [17]. In contrast, a population-based case-control study featuring 324 female RA participants from western Washington and 1,245 healthy controls found that increased protein consumption leads to decreased risk of developing RA, and the association became stronger when examining RF+ cases only [33]. Furthermore, several studies have not found a statistically significant association between dietary protein and risk of developing RA. A nested case-control study using the VIP cohort found no statistically significant association between protein consumption and the risk of developing RA [16]. A prospective cohort study that focused on protein, iron, and meat consumption in the NHS cohort did not find an association between protein intake, including animal and vegetable protein, and the risk of developing RA [31].

Fish

Fish has been hypothesized to reduce the risk of developing RA through its high levels of n-3 polyunsaturated fatty acids (PUFAs), particularly eicosapentaenoic acid (EPA) and docosahexaenoic acid (DHA), which are known to be anti-inflammatory [35]. A population-based case-control study featuring 324 female RA participants from western Washington and 1,245 healthy controls, which used a food frequency questionnaire to examine diet, found that increased consumption of boiled or baked fish reduced the risk of developing RA, and the association became stronger when examining RF+ cases only [33]. However, this trend was not found with other types of fish, such as tuna (including salad and casserole), shellfish, fried fish or fish sandwich, or all fish items (including shellfish) [33]. A serially matched case-control study consisting of 145 cases and 188 healthy controls found no statistically significant association between increased fish consumption and the risk of developing RA [36]. A prospective study featuring the NHS cohort did not find an association between fish consumption and the risk of developing RA [31]. A population-based prospective study featuring the Swedish Mammography Cohort found an association that suggests a reduced risk of developing RA when consuming >1 serving of fish/week versus <1 serving of fish/week (RR 0.71, 95% CI 0.48-1.04) [37]. A nested case-control study using the VIP cohort found no statistically significant association between fish consumption and the risk of developing RA [16]. A case-control study featuring 968 Han Chinese RA patients and 1,037 matched healthy controls did not find a statistically significant association between fish consumption and the risk of developing RA [30]. A case-control study consisting of 1,889 cases and 2,145 healthy controls from a Swedish population found that consuming oily fish one to seven times a week reduced the risk of developing RA (OR 0.8, 95% CI=0.6 -1.0) [38]. Similar results were seen for participants who consumed oily fish one to three times a month [38]. A prospective cohort study featuring the NHS and NHS II cohorts found no significant association between fish intake on RA risk [39].

Results regarding fish consumption and RA risk have either shown a statistically significant association between increased consumption and decreased risk of developing RA or have not been statistically significant. Thus, fish consumption is possibly protective against the risk of developing RA, but more research is needed.

Fats

The role of fats, particularly PUFAs such as EPA and DHA, has been studied in autoimmune diseases, such as RA [40]. A study featuring 145 RA patients and 188 healthy controls found an inverse association between increased olive oil consumption and risk of developing RA (OR: 0.39; chi-square: 4.28; P=0.03) [36]. A nested case-control study using the VIP cohort, which looked at the impact of diet and alcohol consumption on the risk of developing RA, found no significant association between fat intake, including fatty acids such as arachidonic acid and long-chain omega-3 acids, and the risk of developing RA [16]. A study using the EPIC-Norfolk that examined the association of red meat and other dietary components and the risk of developing IP showed that increased intake of polyunsaturated and monounsaturated fats was weakly protective against developing IP, while increased intake of saturated fat was associated with a slight increase in the risk of IP [17]. A population-based prospective study featuring the Swedish Mammography Cohort, which assessed the association between dietary long-chain n-3 PUFAs and the incidence of RA in middle-aged and older women using a self-administered food-frequency questionnaire, found that consuming more than 0.21 g/day (lowest quintile) of dietary long-chain n-3 PUFAs was associated with a 35% decreased risk of developing RA (RR=0.65; 95% CI 0.48 to 0.90) [37]. The study also found that long-term consumption of more than 0.21 g/day of dietary long-chain n-3 PUFAs was associated with a 52% decreased risk of developing RA (95% CI 29% to 67%) [37]. A population-based case-control study featuring 324 female RA participants from western Washington and 1,245 healthy controls found no significant association between increased fat intake, including omega-3 fatty acids, and the risk of developing RA [33]. A case-control study featuring 500 patients with RA and 500 healthy controls found that increased monthly consumption of solid oil increases risk of developing RA (OR=2.29, CI: 1.57-3.34) [28]. A prospective cohort study featuring the NHS and NHS II cohorts found no significant association between marine omega-3 fatty acid intake on RA risk [39].

Results regarding fat consumption and risk of RA have been mixed but increased consumption of unsaturated fatty acids, specifically monounsaturated fatty acids (MUFAs) and PUFAs, is likely protective against developing RA. Further research is needed.

Flavonoids

Increased flavonoid intake, an antioxidant found in plants, has been shown to decrease the risk of chronic disease [41]. One study that examined the relationship between flavonoid intake and risk of chronic disease analyzed the dietary intakes of 10,054 men and women and found that increased kaempferol, a flavonoid, consumption increases the risk of developing RF+ RA only (RR: 1.91; 95% CI: 1.01, 3.62; P=0.05) [41]. No statistically significant association between increased quercetin, myricetin, hesperetin, or naringenin consumption and RA risk was found [41]. However, it is important to note that there was only one study found that examined the influence of flavonoids on the risk of developing RA. 

Sugary Drinks

Sugary drinks have recently been considered a possible risk factor for developing RA, especially due to their indications in other chronic inflammatory conditions [42]. Increased consumption of beverages with excess free fructose has been found to be associated with arthritis in US adults 20-30 years of age [43]. A prospective cohort study using the NHS and NHS II cohorts, which examined the consumption of sugar-sweetened soda, found a 63% increased risk of developing seropositive (but not seronegative) RA in women who consumed >1 serving of sugar-sweetened soda/day in comparison with women who do not drink sugar-sweetened soda or consume <1 serving/month (HR: 1.63; 95% CI: 1.15, 2.30; P-trend=0.004) [44]. This trend became even more significant when analyzing women with RA onset at >55 years of age (HR: 2.64; 95% CI: 1.56, 4.46; p-trend=0.0001) [44]. There was no significant association between diet soda consumption and seropositive or seronegative RA [44]. 

While more research must be done, increased consumption of sugary drinks appears to increase the risk of developing RA.

Sodium

Sodium is a chemical element that is part of commonly consumed table salt and is also naturally occurring in many fruits and vegetables. Thus, it is a significant component of people's daily diet. Sodium intake has been shown to be a possible environmental risk factor for developing autoimmune diseases, such as RA [45]. A cross-sectional, case-control study featuring the SUN (Seguimiento Universidad de Navarra) cohort found a significant association between self-reported RA cases and being in the highest quartile of sodium consumption (fully adjusted odds ratio 1.5; 95% CI 1.1-2.1, P-trend=0.02) [46]. Currently, there are few studies regarding the association between sodium and RA risk, but increased sodium consumption is a potential risk factor for developing RA.

Vegetables

The role of vegetables, which are high in antioxidants, on RA risk has also been studied. A study using the Iowa Women’s Health Study cohort, which analyzed antioxidant micronutrients and the risk of developing RA, found an association between increased cruciferous vegetable (e.g., broccoli) intake and decreased risk of developing RA [20]. A nested case-control study using the VIP cohort, which looked at the impact of diet and alcohol consumption on the risk of developing RA, found no significant association between dietary items (including vegetables) and the risk of developing RA [16]. A case-control study featuring 968 Han Chinese RA patients and 1,037 matched health controls found no significant association between increased vegetable consumption and the risk of developing RA [30]. Furthermore, a study using the EPIC-Norfolk that examined the association of red meat and other dietary components and the risk of developing IP showed no significant association between vegetable consumption and developing IP [17]. 

A population-based case-control study featuring 324 female RA participants from western Washington and 1,245 healthy controls, which used a food frequency questionnaire to examine diet, did not find a significant association between vegetable consumption and the risk of developing RA [33]. A study featuring 145 RA patients and 188 healthy controls found an inverse association between increased cooked, but not raw, vegetable consumption and risk of developing RA (OR: 0.24, chi-square: 10.48; P=0.001) [36]. A case-control study that focused on dietary intake which featured 968 Han Chinese RA patients and 1,037 matched healthy controls found that increased consumption of mushrooms decreases the risk of developing RA (aOR=0.669; 95% CI=0.518-0.864, P=0.002), but there is no significant association between bean consumption and RA risk [30].

While more research still needs to be done, it appears that total vegetable consumption does not have a significant impact on the risk of developing RA. However, cruciferous vegetable and mushroom consumption may prevent the development of RA.

Fruit

Fruits, which are rich in antioxidants such as flavonoids, have been shown to reduce pain and inflammation in arthritis [47]. A nested case-control study using the VIP cohort, which looked at the impact of diet and alcohol consumption on the risk of developing RA, found no significant association between dietary items (including fruit) and the risk of developing RA [16]. A case-control study that focused on the dietary intake that featured 968 Han Chinese RA patients and 1,037 matched healthy controls found that increased consumption of citrus fruits decreases the risk of developing RA (aOR=0.990; 95% CI=0.981-0.999, P=0.04) [30]. A nested case-control study featuring the EPIC-Norfolk cohort found an association between decreased fruit consumption and increased risk of developing IP [21]. A population-based case-control study featuring 324 female RA participants from western Washington and 1,245 healthy controls, which used a food frequency questionnaire to examine diet, did not find a significant association between fruit consumption and the risk of developing RA [33]. A study using the Iowa Women’s Health Study cohort found an association between increased fruit intake and reduced risk of developing RA (RR=0.72, 95% CI: 0.46, 1.12; p-trend=0.13) [20]. A population-based case-control study featuring 324 female RA participants from western Washington and 1,245 healthy controls, which used a food frequency questionnaire to examine diet, found no association between fruit consumption and risk of developing RA [33]. A study featuring the Women’s Health Initiative Observational Study prospective cohort, which features 76,853 older women, did not find a significant association between coffee consumption, including amount, caffeinated versus decaffeinated, and filtered/unfiltered, and risk of developing RA.

In general, there does not appear to be a significant association between the risk of RA and fruit consumption. However, fruit consumption may prevent the risk of developing RA.

Dairy

Dairy products have been shown to be weakly anti-inflammatory [48]. A case-control study that focused on dietary intake which featured 968 Han Chinese RA patients and 1,037 matched healthy controls found that increased consumption of dairy products decreases the risk of developing RA (aOR=0.921; 95% CI: 0.867-0.977, P=0.006) [30]. A population-based case-control study featuring 324 female RA participants from western Washington and 1,245 healthy controls found no significant association between dairy consumption and risk of developing RA [33]. A case-control study featuring 500 patients with RA and 500 healthy controls found that increased monthly consumption of full-fat milk increases risk of developing RA (OR=1.01, CI 1.003-1.03) [28]. A study using the cohort EPIC-Norfolk that examined the association of red meat and other dietary components and the risk of developing IP found a weak association between increased dairy consumption and RA risk [16]. While more research needs to be done, dairy consumption may increase RA risk.

Elements

A study examining dietary risk factors and developing inflammatory arthritis featuring the EPIC-Norfolk population found no significant association between iron consumption and the risk of developing IP [17]. A prospective cohort study that focused on protein, iron, and meat consumption in the NHS cohort did not find an association between iron intake and risk of developing RA [31]. A population-based case-control study featuring 324 female RA participants from western Washington and 1,245 healthy controls did not find a significant association between iron, calcium, and phosphorus intake with risk of developing RA [33]. A nested case-control study featuring the EPIC-Norfolk cohort found no significant association between dietary selenium intake and the risk of developing IP [21]. 

There does not appear to be a significant association between iron, calcium, phosphorus, and selenium intake and RA risk.

Overall Dietary Pattern

The overall dietary pattern in patients with RA has long been a topic of interest. Patients may change their diets after being diagnosed with RA to manage symptoms, such as with the Mediterranean diet, but not many studies have shown the influence of overall dietary pattern as a risk factor for developing RA [49]. Another study featuring the NHS cohort that focused on dietary quality, as evaluated by AHEI-2010 components, found that a healthier diet was associated with reduced RA risk, especially seropositive RA risk, in women <55 years of age [11]. A “healthier diet” includes increased fruit, vegetable, whole grain, nut, long chain, polyunsaturated fat, moderate alcohol consumption, and decreased sugar-sweetened beverages, red/processed meat, trans fat, sodium consumption [11]. A study featuring the NHS and NHS II cohorts that focused on inflammatory dietary patterns using the Empirical Dietary Inflammatory Pattern (EDIP) and risk of RA found that increased EDIP was associated with increased overall RA risk in women <55 years of age (HRs (95% CIs) across EDIP quartiles 1.14 (0.86-1.51), 1.35 (1.03-1.77), and 1.38 (1.05-1.83; p-trend=0.01)), but significance was reduced after adjusting for BMI categories (<25, 25-29.9, ≥30 kg/m^2^) [50]. Increased EDIP was also significantly associated with the risk of seropositive RA in women <55 years of age (p-trend=0.04), but not >55 years of age (p-trend=0.03) [50]. A prospective cohort study featuring the NHS and NHS II cohorts did not find a significant association between a Mediterranean diet and the risk of developing RA [51].

It appears that overall dietary quality may play a role in increased RA risk, especially unhealthy and inflammatory diets. Further studies need to be done on the influence of the Mediterranean diet on RA risk.

Limitations:

While care was taken to minimize the impact of variation across dietary item categorization, RA serostatus, and study design, these may play a role in why there was no significant influence found in certain dietary factors. Further studies need to be completed to make definitive conclusions about these dietary factors. Another limitation of this study is that it is a narrative review.

## Conclusions

The exact etiology of RA is still being studied. Diet is a modifiable environmental factor that may play a role in developing the disease. There have been multiple studies in recent years that have focused on diet as a risk factor for RA, but results have often been mixed or inconclusive. This narrative review serves to clarify the impact of individual dietary factors on developing RA. 

When the variations across studies in the categorization of each dietary item, RA serostatus, methods of data collection, and cohorts studied were considered, a decreased risk of developing RA was associated with moderate alcohol consumption (one drink a day for women; two drinks a day for men). It is important to remember that increased alcohol intake can also lead to complications such as liver and cardiovascular disease. Thus, the risks and benefits of alcohol consumption should be weighed when counseling patients about increasing alcohol consumption and the risk of developing RA. Several studies in this article also showed that increased β-cryptoxanthin (a carotenoid) consumption may have protective effects against developing RA. This may be applicable clinically by discussing increasing the intake of foods rich in β-cryptoxanthin with patients, such as oranges.

From the studies examined in this article, it appears that the increased risk of developing RA was associated with increased meat, sugary drink, and sodium consumption, and poor overall diet.

There was no significant influence in developing RA found with consumption of caffeine, meat, carbohydrates, total protein, fish, fat, sugary drinks, sodium, vegetables, fruit, dairy, elements, and overall dietary pattern. However, it is important to note that there were limited studies for certain dietary items, such as flavonoids, where only one study was found. Thus, more studies need to be done to draw further conclusions about dietary items that had limited studies. 

Overall, specific dietary elements and their influence on RA risk is a promising topic, as it is a modifiable risk factor. Significant findings may be helpful in counseling patients, preventing the development of RA, and improving our overall understanding of the pathophysiology of the disease. 
